# 25 years of experience with transjugular intrahepatic portosystemic shunt (TIPS): changes in patient selection and procedural aspects

**DOI:** 10.1186/s13244-022-01216-5

**Published:** 2022-04-13

**Authors:** Laura Büttner, Annette Aigner, Lisa Pick, Josefine Brittinger, Christian J. Steib, Georg Böning, Florian Streitparth

**Affiliations:** 1 Department of Radiology, Charité –Universitätsmedizin Berlin, corporate member of Freie Universität Berlin and Humboldt-Universität zu Berlin, Charitéplatz 1, 10117 Berlin, Germany; 2 Institute of Biometry, Charité – Universitätsmedizin Berlin, corporate member of Freie Universität Berlin and Humboldt-Universität zu Berlin, Charitéplatz 1, 10117 Berlin, Germany; 3 Medical Clinic II, LMU Munich, Marchioninistr. 15, 81377 Munich, Germany; 4 Department of Radiology, LMU Munich, Marchioninistr. 15, 81377 Munich, Germany

**Keywords:** Transjugular intrahepatic portosystemic shunt (TIPS), Portal hypertension, Variceal bleeding, Ascites, Hepatic encephalopathy

## Abstract

**Background:**

TIPS is an established treatment for portal hypertension. The aim was to analyze how patient selection for TIPS implantation and procedural aspects have changed over 25 years. Routinely collected demographic, clinical, laboratory, and procedural data of 835 patients treated with TIPS in a single center were used. Time trends over the observational period from 1993 to 2018 were retrospectively analyzed. Descriptive statistical analysis was performed.

**Results:**

The most common indication for TIPS implantation has changed significantly from secondary prevention of variceal hemorrhage in the early years to treatment of recurrent ascites. During the observation period, increasingly more severely ill patients became TIPS candidates. There was little change in MELD scores over this period (in total median 13.00; IQR 10.00–18.00). The proportion of patients with Child–Pugh C cirrhosis increased. The most frequent underlying diseases in total were alcohol-related liver disease (66.5%) and viral hepatitis (11.9%). However, shares of cryptogenic liver cirrhosis, autoimmune hepatitis, and NASH increased over time. The proportion of patients post liver transplant also increased. While bare metal stents were standard in the past, use of covered stents increased. The success rate of TIPS (defined by successful implantation and a decrease in the portosystemic pressure gradient ≤ 12 mmHg) increased significantly over time. The total success rate according to this definition was 84.9%.

**Conclusion:**

The results of our analysis reflect technical developments in TIPS, especially in terms of stent material and gains in clinical experience, particularly regarding indications and patient selection for TIPS implantation.

## Key points

- During the last decades, technical advances and a gain in clinical experience with TIPS have taken place.
- Bare metal stents are increasingly replaced by covered stents and ultrasound guidance became gold standard in clinical routine.
- The most common indications for TIPS have switched from secondary prevention of variceal hemorrhage to treatment of recurrent ascites.

## Background

Since the end of the 1980s, transjugular intrahepatic portosystemic shunt (TIPS) placement has been increasingly used to treat the symptoms of portal hypertension and is now considered the gold standard among interventional/surgical options [1–5]. During this period, there have been several technical improvements, and clinical experience has increased. Portal hypertension is considered as a portosystemic pressure gradient (PSPG) > 10 mmHg [2]. Clinically, portal hypertension leads to the development of collateral circulation such as esophageal and gastric varices, splenomegaly, ascites, hepatorenal syndrome, hepatopulmonary syndrome, and portopulmonary hypertension [6–8]. The most frequent indications for TIPS in Germany are therapy refractory or recurrent ascites (up to 70%) [9] and secondary prevention of variceal hemorrhage [2, 10]. Other less frequent indications for TIPS are portal hypertensive gastropathy, hepatic hydrothorax, hepatorenal syndrome, and Budd–Chiari syndrome [2].

Patient selection for TIPS is an interdisciplinary decision which takes into account demographic, clinical, laboratory, and echocardiographic parameters, as well as standard scoring systems such as the Child–Pugh score (CPS) and the model for end-stage liver disease (MELD) [2]. In addition to careful patient selection, active follow-up surveillance is recommended to ensure prevention, early detection, and treatment of complications and TIPS dysfunction [10].

The aim of this study was to analyze how patient selection for TIPS implantation and procedural aspects have changed over 25 years.

## Material and methods

### Study population

This retrospective analysis included 835 patients who underwent TIPS treatment at our center from June 1993 to December 2018. Keyword searches in our department´s archive initially identified 1195 cases. A total of 360 cases had to be excluded for various reasons including ex domo TIPS implantation or double hits because of different spellings of patient names. Follow-up was defined until the end of the observation period in 2018, or, if occurred before: death, liver transplantation (bridge to transplant), the last recanalization attempt (for non-recanalizable TIPS thrombosis), iatrogenic TIPS occlusion (e.g., in patients with right heart failure due to increased preload), or the last patient contact within the observation period (lost to follow-up/end of observation). We collected (i) demographic data, (ii) clinical data, (iii) laboratory, and (iv) procedural parameters. No distinction was made between refractory and recurrent ascites since a clear assignment to either category was not possible in this retrospective study. The study was approved by the responsible ethics committee (EA4/085/17).

### Technique of TIPS

Indications for TIPS implantations were established by interdisciplinary case review of hepatologists, radiologists, and surgeons. Contraindications to TIPS implantation have changed considerably in recent years. For better comparability, we here used the current recommendation of Schultheiß et al. to classify contraindications [2]. TIPS was performed as described before [2]. The PSPG was measured between the portal vein and either the inferior vena cava/hepatic vein or the right atrium, which, due to the retrospective study design, could not be determined with certainty. Of note, Mura et al. reported that right atrial pressure may not be adequate to calculate portal pressure gradient in cirrhosis [11]. A reduction of the portosystemic pressure gradient (PSPG) ≤ 12 mmHg was defined as successful TIPS (primary success). Failure to achieve this goal with the initial TIPS procedure in conjunction with a clinically suboptimal symptom reduction was an indication for revision. In some patients, a formally sufficient pressure reduction was achieved (e.g., by further dilatation of the tract) during the re-intervention (secondary success). Patients were monitored in the intensive care unit for 24 h before transfer to the normal ward. Follow-up intervals in our hospital are day 1 (ultrasound of TIPS flow profile), months 3, 6, and 12 during the first year, then every 12 months. In patients with ascites, paracentesis directly before TIPS became established in recent years to detect bleeding as a color change from yellow (ascites) to red (blood).

### Statistical analysis

To reveal time trends over the observational period from 1993 to 2018, a descriptive statistical analysis was performed. We report absolute and relative frequencies for categorical variables and medians along with interquartile ranges for continuous variables—for the total study population and for 5-year periods. To display trends over time more clearly, locally weighted scatterplot smoothing (LOESS) estimates are plotted along with 95% confidence intervals. Statistical analysis was performed using R (R Core Team) including additional packages for data handling and plotting [12, 13].

## Results

### Demographic patient data

Over the 25 years of observation, the male-to-female ratio remained nearly constant—two thirds of patients were male (66.9%). Average age increased slightly (median of 53 years in 1993–1997 vs 58 years in 2013–2018).

### Clinical patient data

**Fig. 1:**
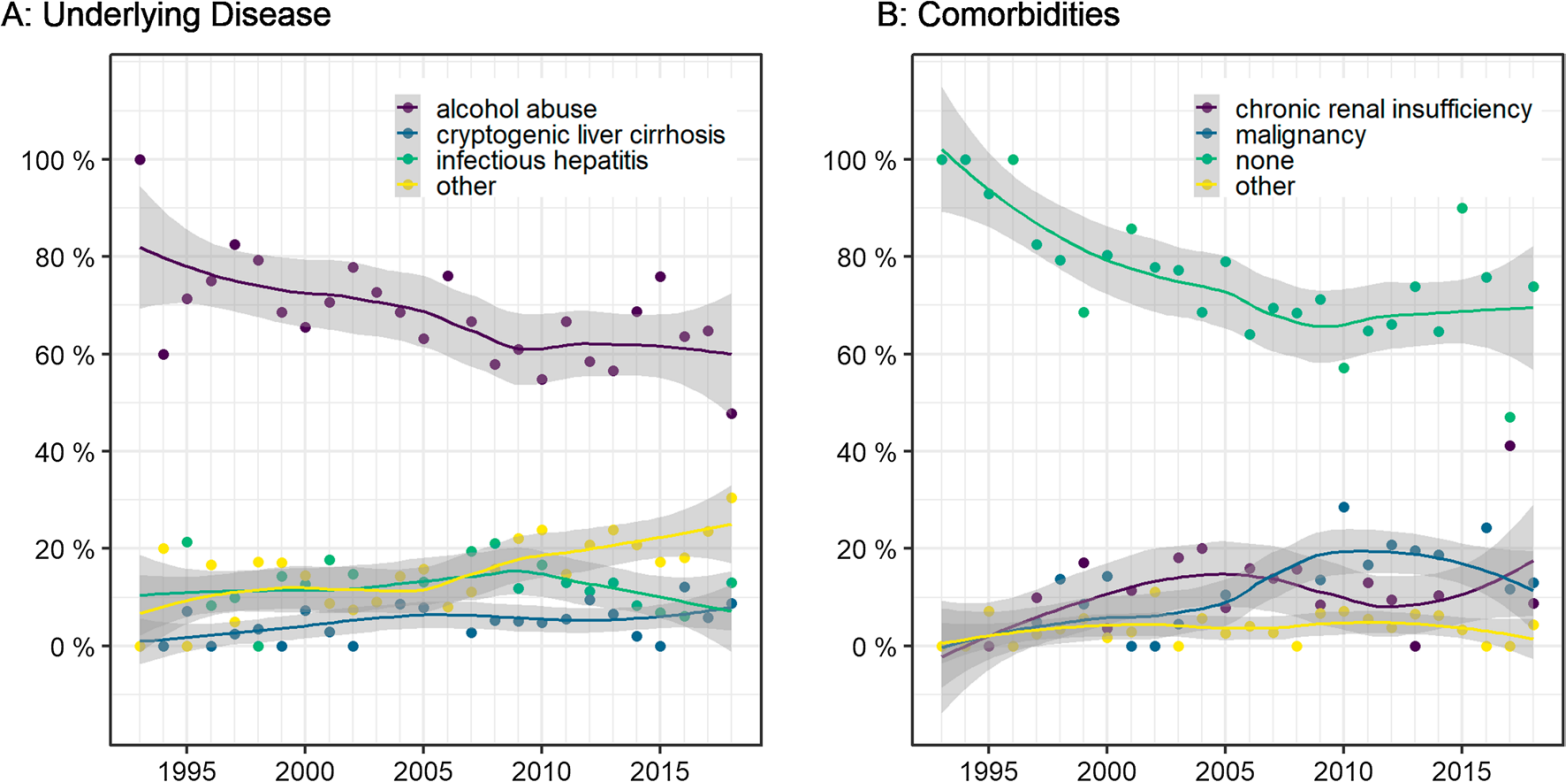
Clinical data by year. **a** Underlying disease. Share of the three most frequent underlying diseases over the observation period. For a summary of all underlying diseases see appendix. **b** Comorbidities. The proportion of patients with comorbidities increased over time

The most frequent underlying diseases were alcohol abuse (66.5%), hepatitis (11.9%), cryptogenic liver cirrhosis (5.3%), and combined hepatitis and alcohol abuse (3.0%). The proportion of patients with alcohol dependence decreased in favor of patients with other underlying diseases, such as autoimmune hepatitis (1.6%), primary biliary cholangitis (PBC) (2.2%), primary sclerosing cholangitis (PSC) (1.1%), Budd–Chiari syndrome (2.6%), nonalcoholic steatohepatitis (NASH) (1.6%), and drug-related cirrhosis (1.2%) (see Fig. 1).

**Fig. 2:**
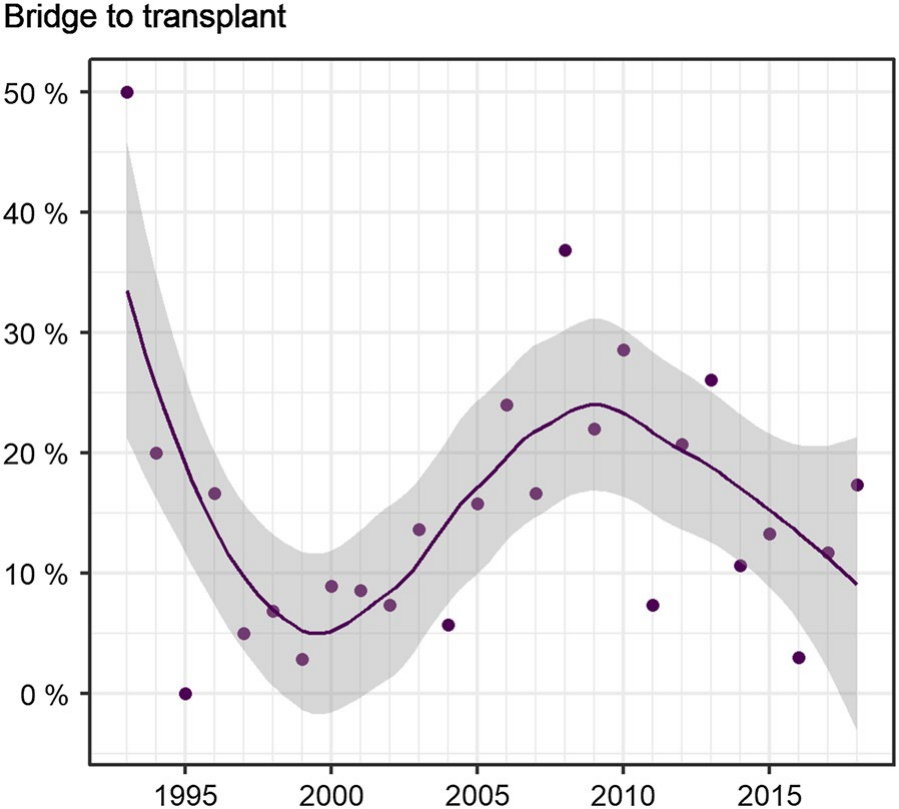
Bridge to transplant. Share of TIPS implantations performed in patients waiting for a liver transplant (14.0% of total study population (*n* = 117)). Note fluctuation over the course of time

The proportion of patients with comorbidities increased over time, from 21.0% initially to 26.8% at the end of the observation period (see Fig. 1). The most frequent comorbidities were intra- and extrahepatic malignancies (*n* = 108, 12.9%), chronic renal insufficiency (*n* = 81, 9.7%), and coagulopathy (*n* = 21, 2.6%). The proportion of patients with liver transplant increased during the observation period (0.0% in 1993–1997 vs 4.6% in 2013–2018). A total of 14.0% (*n* = 117) of TIPS implantations were performed as bridge to transplant (see Fig. 2).

**Fig. 3:**
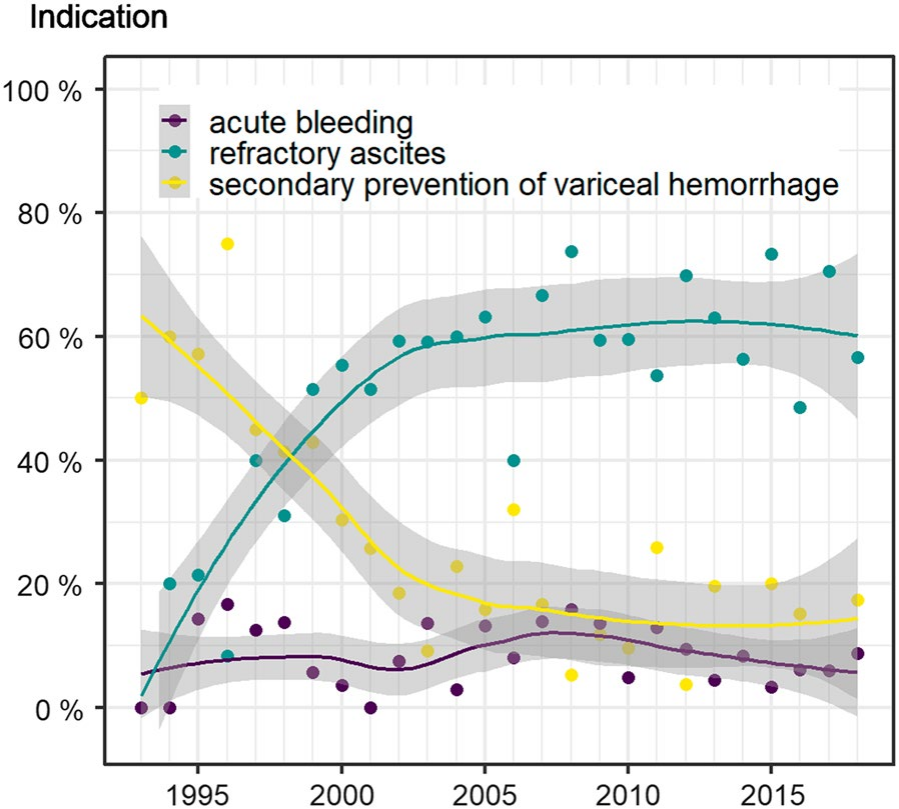
TIPS indications. TIPS indications changed significantly over 25 years

Indications for TIPS implantation shifted significantly during the observation period. In the beginning, secondary prevention of variceal hemorrhage was the main indication; this was replaced by treatment of refractory ascites (see Fig. 3). Overall, the most frequent indications were refractory ascites (55.6%), secondary prevention of variceal hemorrhage (22.0%), acute bleeding event in refractory variceal hemorrhage (8.6%), and a combination of variceal hemorrhage and acute bleeding (5.7%). Rare indications included acute bleeding from gastric varices (1.3%) or hepatorenal syndrome (2.9%).

The majority of patients had no contraindication to TIPS implantation (67.5%); 24.8% of interventions were performed despite relative contraindication (e.g., bilirubin 3-5 mg/dl, INR > 5, or cystic liver). Only a few TIPS implantations were performed despite absolute contraindications (7.7%), e.g., in cases of acute hemorrhage impossible to control otherwise.

**Fig. 4:**
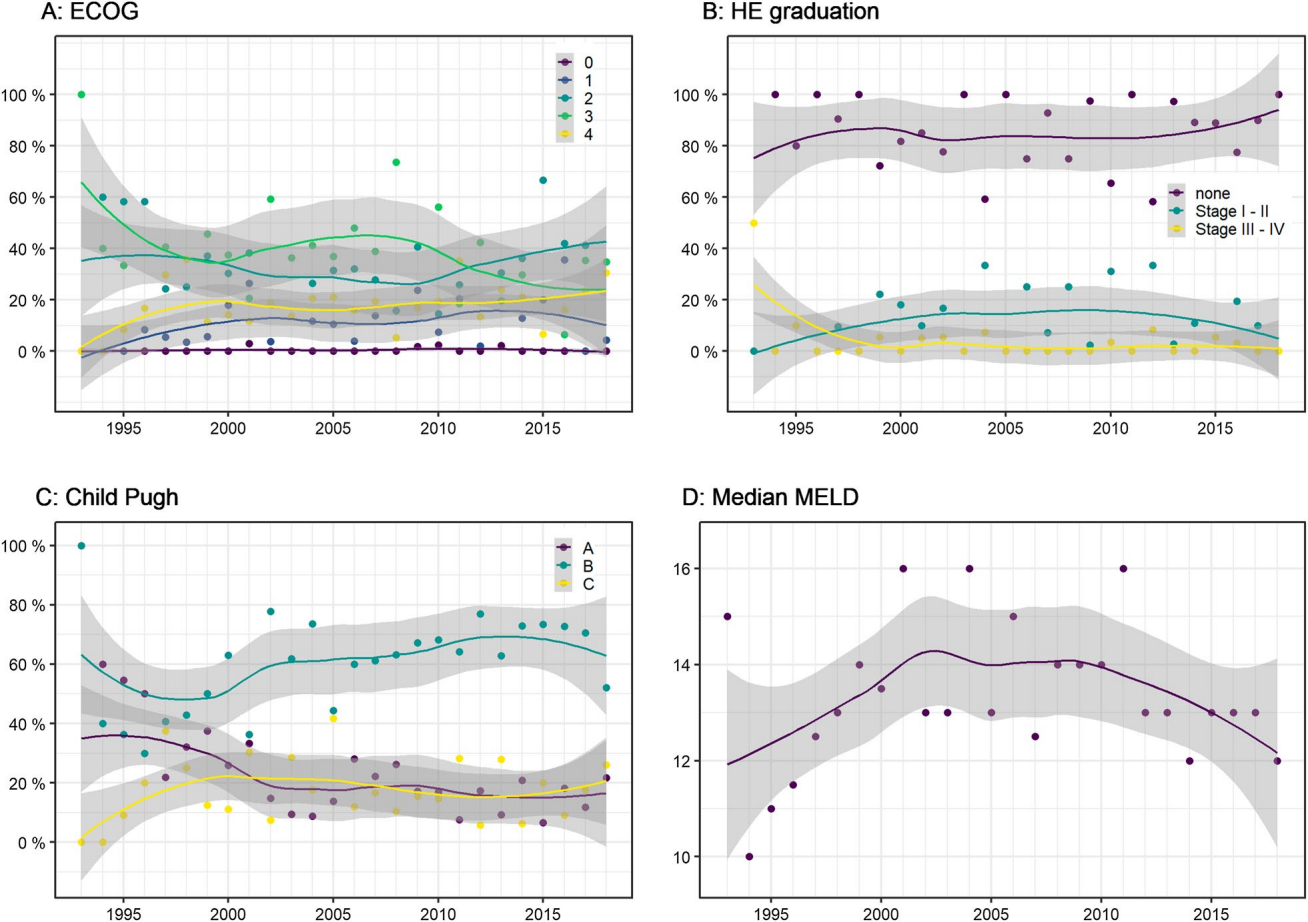
Clinical and laboratory parameters. **a** Eastern Cooperative Oncology Group (ECOG) score. Proportion of patients classified according to ECOG score during the observation period. ECOG: 0—asymptomatic, 1—symptomatic but completely ambulatory, 2—symptomatic, < 50% in bed during the day, 3—symptomatic, > 50% in bed, but not bedbound, 4—bedbound, 5—death [45]. **b** Hepatic encephalopathy before TIPS. Proportion of patients with HE classified according to the West Haven Criteria over the observation period. I: mild symptoms, e.g., loss of sleep and shortened attention span. II: moderate symptoms, e.g., memory loss and slurred speech. III: severe symptoms, e.g., personality changes, confusion, and extreme lethargy. IV: a loss of consciousness and coma [46]. **c** Child–Pugh score. Child–Pugh score over the observation period. **d** MELD score. Median MELD score over the observation period. Three patients had a MELD score over 40, all of them with CPS C and ECOG score 3; one of them underwent TIPS in an emergency setting

Performance status was assessed using the ECOG score (see Fig. 4). Only 4 patients (0.5%) had an ECOG score 0. The majority of patients had either ECOG 2 (*n* = 275, 33.5%) or ECOG 3 (*n* = 277, 33.7%). The share of patient with ECOG 4 slightly increased over time (in total *n* = 158, 19.2%). The proportion of patients with ECOG 1 fluctuated over the observation period; their total proportion was 13.1% (*n* = 108). The majority of patients had no hepatic encephalopathy (HE) before TIPS implantation (90.2%). Only a few patients had stage I–II (8.4%) and stage III–IV HE (1.3%). The proportions of these two HE categories were constant during the observation period (see Fig. 4).

### Laboratory parameters

Over the decades, the proportion of patients with Child–Pugh score (CPS) C increased, while the proportion of patients with CPS A decreased. Overall, the majority of patients had CPS B (61.6%, *n* = 495). CPS A (19.9%, *n* = 160) and CPS C (18.4%, *n* = 148) were less frequent. The MELD score was rather constant throughout our study period with a median MELD score of 13 (IQR 10.00–18.00) (see Fig. 4).

### Procedural parameters

**Fig. 5:**
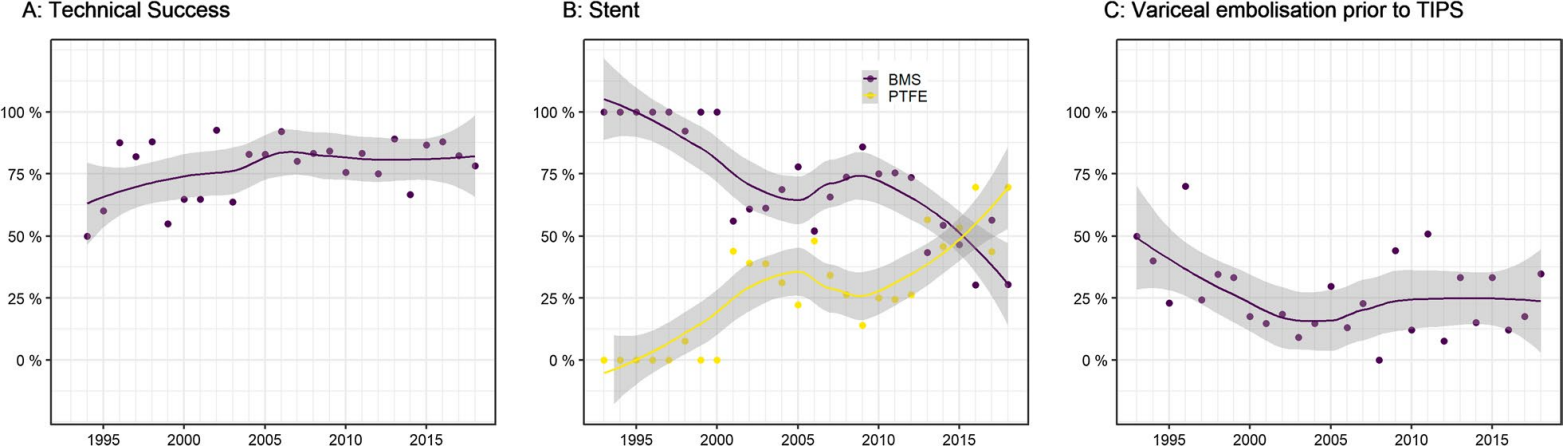
Procedural data. **a** Trend in the technical success rate over 25 years. Primary technical success rate in total: 77.9%, secondary technical success rate in total: 84.9%. **b** Stent type use over time. **c** Variceal vein embolization prior to TIPS

The success rate (defined by PSPG) increased over time and has been above 80% since 2010 (see Fig. 5); primary success rate was 77.9%, secondary success rate was 84.9%. In the beginning, only bare metal stents (BMS) were used. Since 2010, covered stents became used more frequently. In 24.7% of cases, endoscopic variceal vein embolization was performed by gastroenterologists before TIPS implantation; in 3.7%, embolization was performed peri-interventionally by the interventional radiologist. During the observation period, both pre-interventional and peri-interventional variceal embolizations decreased. 71.6% of TIPS implantations were performed without variceal embolization (see Fig. 5). The proportion of patients with ascites also increased during the last few years. Overall, 80.6% of patients had ascites before TIPS implantation. Over time, paracentesis before TIPS became more common: 30.0% in 1993–1997 vs 78.1% in 2013–2018. Overall, paracentesis was performed before 68.5% of interventions. The share of patients with pre-interventional anticoagulation/antiplatelet treatment increased over the observation period (total of 4.9%). The most common regimen was 100 mg acetylsalicylic acid (2.4%). If there were no bleeding complications, post-interventional heparin for anticoagulation was given for a short-term period of up to 3 days in 89.7%. The proportion of elective TIPS implantations and emergency TIPS implantations was stable over the observation period. The vast majority was elective (*n* = 747, 89.5%), and there were only 88 emergency implantations in this period (10.5%). The majority of TIPS implantations was performed with analgosedation (90.0%), but their proportion decreased during the observation period in favor of general anesthesia (10.0% in total).

In the majority of interventions, the right liver vein was used for access (89.6%, *n* = 657), rarely the middle or left hepatic vein (Table 1). In the early days of TIPS implantation, blind punctures were relatively common (1993–1997: 28.9%) and then decreased (2013–2018: 19.2%). Puncture of the right portal vein using ultrasound guidance was most common (*n* = 565, 75.1%). The proportion of ultrasound-guided punctures also continued to increase over the years (1993–1997: 55.3% vs. 2013–2018: 75.6%). 17% of TIPS implantations were performed under exclusive fluoroscopy using anatomical landmarks for orientation (*n* = 140, 18.6%). Other navigation techniques were seldomly used (see Table 1).

**Table 1:** Procedural data (for the total study period and 5-year periods)

	1993–1997 (*n* = 73)	1998–2002 (*n* = 182)	2003–2007 (*n* = 156)	2008–2012 (*n* = 227)	2013–2018 (*n* = 197)	Total (*n* = 835)
**TIPS implantation setting**						
Emergency TIPS implantation	11 (15.1%)	11 (6.0%)	19 (12.2%)	26 (11.5%)	21 (10.7%)	88 (10.5%)
Elective TIPS implantation	62 (84.9%)	171 (94.0%)	137 (87.8%)	201 (88.5%)	176 (89.3%)	747 (89.5%)
**Anesthesia**						
Analgosedation	61 (92.4%)	162 (92.6%)	135 (93.1%)	201 (88.9%)	152 (85.4%)	711 (90.0%)
General anesthesia	5 (7.6%)	13 (7.4%)	10 (6.9%)	25 (11.1%)	26 (14.6%)	79 (10.0%)
Ascites before TIPS	45 (61.6%)	133 (73.5%)	131 (84.5%)	192 (84.6%)	170 (86.7%)	671 (80.6%)
Ascites puncture/drainage before TIPS	18 (30.0%)	106 (61.3%)	102 (68.0%)	166 (77.2%)	143 (78.1%)	535 (68.5%)
**Anticoagulation before TIPS**						
None	66 (98.5%)	154 (96.9%)	141 (95.3%)	184 (93.4%)	156 (94.0%)	701 (95.1%)
ASS	1 (1.5%)	2 (1.3%)	3 (2.0%)	6 (3.0%)	6 (3.6%)	18 (2.4%)
Heparin	0 (0.0%)	2 (1.3%)	2 (1.4%)	3 (1.5%)	1 (0.6%)	8 (1.1%)
Other anticoagulation	0 (0.0%)	1 (0.6%)	2 (1.4%)	4 (2.0%)	3 (1.8%)	10 (1.4%)
**Anticoagulation after TIPS**						
None	6 (15.8%)	36 (25.5%)	12 (8.5%)	9 (4.5%)	4 (2.1%)	67 (9.5%)
Heparin	31 (81.6%)	103 (73.0%)	129 (90.8%)	189 (95.0%)	183 (97.3%)	635 (89.7%)
Other anticoagulation	1 (2.6%)	2 (1.4%)	1 (0.7%)	1 (0.5%)	1 (0.5%)	6 (0.8%)
**Navigation technique**						
Fluoroscopy only with landmarks	11 (28.9%)	20 (13.0%)	20 (13.8%)	52 (23.4%)	37 (19.2%)	140 (18.6%)
Fluoroscopy and Ultrasound	21 (55.3%)	123 (79.9%)	117 (80.7%)	158 (71.2%)	146 (75.6%)	565 (75.1%)
Direct portography	2 (5.3%)	3 (1.9%)	4 (2.8%)	12 (5.4%)	3 (1.6%)	24 (3.2%)
Indirect portography	4 (10.5%)	2 (1.3%)	1 (0.7%)	0 (0.0%)	0 (0.0%)	7 (0.9%)
CO2-Angiography	0 (0.0%)	5 (3.2%)	2 (1.4%)	0 (0.0%)	0 (0.0%)	7 (0.9%)
Cone-beam CT	0 (0.0%)	0 (0.0%)	0 (0.0%)	0 (0.0%)	6 (3.1%)	6 (0.8%)
Other navigation techniques	0 (0.0%)	1 (0.6%)	1 (0.7%)	0 (0.0%)	1 (0.5%)	3 (0.4%)
**Target liver vein**						
Right liver vein	36 (90.0%)	129 (86.6%)	116 (83.5%)	202 (93.5%)	174 (92.1%)	657 (89.6%)
Middle liver vein	3 (7.5%)	18 (12.1%)	20 (14.4%)	9 (4.2%)	14 (7.4%)	64 (8.7%)
Left liver vein	1 (2.5%)	1 (0.7%)	3 (2.2%)	1 (0.5%)	1 (0.5%)	7 (1.0%)
Variant	0 (0.0%)	1 (0.7%)	0 (0.0%)	4 (1.9%)	0 (0.0%)	5 (0.7%)

Other anticoagulation before TIPS: clopidogrel, marcumar, combination. Other anticoagulation after TIPS: ASS, clopidogrel, marcumar, combination. Other navigation techniques: cone-beam CT, CT, MRI, combination, CO2 angiography, indirect portography

## Discussion

This retrospective study characterizes 835 patients treated with TIPS in a single center between 1993 and 2018. Demographic, clinical, laboratory, and procedural data were evaluated to reveal changes in patient selection and procedural aspects over 25 years of experience with the TIPS procedure.

### Demographic patient data

Over 25 years, there were no discernible trends regarding the male-to-female ratio. The male surplus (67%) of patients is consistent with the epidemiology of liver cirrhosis in industrialized countries and can be explained by the higher incidence of chronic alcohol abuse in men [14]. Patient age was also relatively constant in our patient population.

### Clinical patient data

The leading underlying disease for cirrhosis was alcohol abuse, but its proportion decreased during the study period, while other conditions such as NASH became more common. Over time, the proportion of patients without comorbidities declined, indicating that treatment of patients with more advanced disease became more common in recent years. This is also reflected in the increasing ECOG scores of treated patients, which was especially apparent after 2005.

A slight increase in patients with liver transplant was observed since 2005. Yet the number of liver transplants in Germany is falling [15]. The increase may be due to the fact that patients in need of repeat transplantation, e.g., because of organ failure, have to wait long for a new transplant or have no chance to get a second transplant; those patients might get TIPS as bridging to transplant. There were a total of 117 patients who underwent TIPS implantation as bridging to liver transplantation. Studies show that TIPS improves waiting time survival and thus increases the possibility of curative therapy without having a negative effect on post-transplant survival [16]. Currently, there is still a lack of valid data regarding TIPS before transplant. The fluctuations can possibly be explained by what is known as the “MELD purgatory” [17]. Patients with hepatitis C should receive therapy, if the virus is eradicated, liver function improves and the MELD score decreases, but this leads to patients moving further down the transplant waiting list [17]. The same can be assumed for successful TIPS implantation; a lower MELD score after the intervention indicates less urgency for a liver transplantation.

In the early years of TIPS, the most common indication was secondary prevention of variceal hemorrhage. Since the early 2000s, endoscopic band ligation (EBL) has been increasingly used for this indication with studies indicating an advantage over TIPS [18]. National and international guidelines recommend TIPS for secondary prophylaxis when first-line therapy fails [2]. This recommendation is based on the observation that TIPS implantation is more effective in reducing the recurrent bleeding, but also increases the risk of HE while overall survival remains unchanged [2]. In patients with large and multiple gastric varices, which are difficult to control endoscopically, TIPS is a first-line therapy for secondary hemorrhage prophylaxis [2]. According to national guidelines, coated TIPS should also be considered in patients with recurrent hemorrhage in portal hypertensive gastropathy [19]. Sauerbruch et al. suggest that TIPS should be considered a secondary prophylaxis in younger patients with high portal pressure (> 10 mmHg) and moderate liver dysfunction [20].

Published data indicate that 10–15% of patients with acute variceal hemorrhage require TIPS implantation [21]. 10.5% of TIPS implantations in this subset of patients were performed in an emergency setting, e.g., due to active bleeding. Guidelines consider TIPS a rescue therapy in cases of endoscopically uncontrollable acute variceal hemorrhage (two frustrated endoscopic therapy attempts within 24 h) and hemodynamically relevant recurrent bleeding within five days (secondary failure) [19]. Moreover, so-called early TIPS implantation within 24 to 72 h after endoscopic therapy of acute variceal hemorrhage is recommended in high-risk patients (CPS C, < 14 points) [22]. Hernández-Gea emphasizes the benefits of preemptive TIPS as a treatment of choice in CPS C patients with acute variceal bleeding [23], and Garcia Pagan et al. reported that early use of TIPS significantly reduced treatment failure and mortality in CPS B and C patients [24].Various studies show higher patency rates with coated versus uncoated stents (76 vs. 27%), suggesting that coated stents should be preferred [25, 26].

In recent years, recurrent ascites was the most common indication in our patient population. This is also reflected in the literature—69% of TIPS implantations in Germany are performed for recurrent ascites [9]. For recurrent and refractory ascites, several randomized studies and meta-analyses have shown that TIPS improves ascites control rates compared to conservative treatment with paracentesis [27–29]. A partial or complete response is achieved in up to 76% of patients [2, 29]. TIPS is preferable to repeated large-volume paracentesis in patients with ascites [8].

Only a few patients with HE (stage I–II 8.4%, stage III–IV 1.3%) underwent TIPS implantation in our study population. According to national and international guidelines, TIPS for ascites therapy is usually contraindicated in patients with preexisting chronic HE ≥ II [10]. HE is still a major complication after TIPS implantation, especially in those patients. When bare metal stents (BMS) are used, the HE rate is up to 50%, but the incidence of this complication can be significantly reduced to about 18% when PTFE-covered stents with a smaller diameter (8 mm) are used [8, 30].

The majority of patients were selected respecting absolute and relative contraindications [2]. As mentioned before, contraindications were classified retrospectively using the current recommendations according to Schultheiß et al.[2]. For this reason, it is possible that, during the early years of our study period, some implantations were performed in patients with contraindications according to today's standards. In selected cases with palliative situations and following interdisciplinary consultation, TIPS was performed on the basis of individual decisions despite contraindications.

### Laboratory data

The majority of patients were classified as CPS B. Few patients with CPS A were treated, as these patients often still have sufficient residual liver function and TIPS implantation is therefore not indicated. It is also noticeable that the proportion of patients with a CPS C score increased moderately since 2015, while the proportion of patients with CPS A decreased. Conversely, the MELD score showed little variation over the 25 years (in total median of 13.00; IQR 10.00–18.00). Studies also show that the MELD score appears to be more important in predicting survival, especially in patients with CPS C [7].

### Procedural data

The success rate (defined by a reduction of the PSPG ≤ 12 mmHG) has been above 80% since 2010 (total primary success rate of 77.9%, total secondary success rate of 84.9%). Variations in success rate may be explained by the different levels of training of the interventionalists. Studies suggest that high-level experience is associated with greater stent patency [31]. Success rates may have improved significantly over the 25 years in our patient population mainly because of the introduction of ultrasound guidance [32] and because of experience gained, e.g., through TIPS workshops, as well as improved technical equipment such as wires and catheters. The success rate in our patients is comparable to other studies; a meta-analysis reports a pooled success rate of 86.7% [33]. Tripathi et al. found an even higher success rate of 95% (PPG immediately post-TIPSS of < 12 mmHg or a > 20% reduction in the PPG pre-TIPSS) in 10-year follow-up of 472 patients [34].

In 2003, PTFE-covered stents were licensed and increasingly used for TIPS because of advantages demonstrated in numerous studies, in particular longer patency rates, better sealing to bile ducts, preventions of leakage or fistulas and a survival benefit in patients with refractory and/or recurrent ascites [2]. In Germany, nearly one-third of TIPS implantations are still performed with bare metal stents, most likely because of the twofold to threefold higher costs of covered devices [9]. In our patient population, covered stents were increasingly used since 2010. The current German guidelines also recommend the use of PTFE-covered stents; therefore reimbursement by health insurance companies may increase, helping covered stents to become established as standard [2]. The proportion of pre-interventional paracentesis may also have increased as kind of a learning effect, as it reduces liver mobility and thus contributes to the feasibility and safety of TIPS placement. The proportion of patients with interventional variceal embolization has decreased slightly over the 25 years, possibly due to the therapeutic success of endoscopic procedures and TIPS as described above [2]. Regarding optimal anticoagulation after TIPS implantation, there is no recommendation in the current guidelines [10]. The proportion of interventions performed with analgosedation decreased significantly during the decades of our study period, since an intervention under general anesthesia, if medically justifiable, improves patient comfort.

Regarding the target vein, there was little variability in our cohort over 25 years; in most cases the right liver vein was used (89.6%). Since the early 2000s, at least 75% of punctures of the right portal vein or a right-sided branch were performed with ultrasound guidance. Navigation technology is a dynamic field; in the meantime, cone-beam computed tomography or augmented reality-supported systems are being investigated [35–37].

This study is limited by its retrospective study design. Data from over 25 years were collected, which is also a possible weakness of this study, since documentation standards have changed over time, and data were incomplete in some cases. Our retrospective analysis had no exclusion criteria regarding TIPS indications, severity of underlying disease, secondary diseases, type of TIPS procedure, and stent type. As a result, our patient population was large but heterogeneous compared to other studies [38–40]. However, basic epidemiological data such as age, sex, underlying diseases, as well as the severity of liver disease were comparable to other retrospective studies [41–44].

Overall, the study traces the history of TIPS placement and its development into a standard procedure. Our results illustrate technical advances of the method, such as the introduction of covered stents and improvements in guidance techniques, but also clinical consensus, especially in terms of indications and contraindications, as well as increasing experience of interventional radiologists. All of these factors have helped to optimize the success rate of TIPS. Our data highlight that proper indication and patient selection, in particular, are crucial for good outcome results.

## Conclusion

The results of our analysis reflect the technical developments in TIPS, especially in terms of stent material and gains in clinical experience, particularly regarding indications and patient selection for TIPS implantation.

## Data Availability

The datasets used and/or analyzed during the current study are available from the corresponding author on reasonable request.

## Abbreviations

BMS: Bare metal stent
CPS: Child–Pugh score
DSA: Digital subtraction angiography
ECOG: Eastern Cooperative Oncology Group
EF: Ejection fraction
HCC: Hepatocellular carcinoma
HE: Hepatic encephalopathy
INR: International normalized ratio
IQR: Interquartile range
MELD: Model for end-stage liver disease
NASH: Nonalcoholic steatohepatitis
PBC: Primary biliary cholangitis
PSPG: Portosystemic pressure gradient
PTFE: Polytetrafluorethylene
SBP: Spontaneous bacterial peritonitis.
TIPS: Transjugular intrahepatic portosystemic shunt
